# Comparison of the recovery time of remimazolam besylate and propofol for gastrointestinal endoscopy sedation in elderly patients

**DOI:** 10.7150/ijms.93045

**Published:** 2024-05-13

**Authors:** Hai-yan Chen, Xiao-xi Wang, Yu-gang Lu, Shu-heng Tang, Tong Ding, Jin-Chao Song, Gang Chen

**Affiliations:** 1 Department of Anesthesiology, Sir Run Run Shaw Hospital, School of Medicine, Zhejiang University, Hangzhou, China.; 2 Department of Anesthesiology, Shidong Hospital Affiliated to University of Shanghai for Science and Technology, Shanghai, China.; 3 Department of Anesthesiology, Eastern Hepatobillary Surgical Hospital, Naval Medical University, Shanghai, China.; 4 Department of Anesthesiology, Shanghai Pulmonary Hospital, School of Medicine, Tongji University, Shanghai, China.

**Keywords:** remimazolam, propofol, recovery time, gastrointestinal endoscopy, elderly patients

## Abstract

**Background:** Recovery time is a crucial factor in ensuring the safety and effectiveness of both patients and endoscopy centers. Propofol is often preferred due to its fast onset and minimal side effects. Remimazolam is a new intravenous sedative agent, characterized by its rapid onset of action, quick recovery and organ-independent metabolism. Importantly, its effect can be specifically antagonized by flumazenil. The primary goal of this study is to compare the recovery time of remimazolam besylate and propofol anesthesia during endoscopic procedures in elderly patients.

**Methods:** 60 patients aged 65-95 years who underwent gastrointestinal endoscopy were randomly and equally assigned to two groups: the remimazolam group (Group R) and the propofol group (Group P). The primary measure was the recovery time, defined as the time from discontinuing remimazolam or propofol until reaching an Observer's Assessment of Alertness and Sedation scale (OAA/S) score of 5 (responds readily to name spoken in normal tone). The time required to achieve an OAA/S score of 3 (responds after name spoken loudly or repeatedly along with glazed marked ptosis) was also recorded and compared.

**Results:** The recovery time for Group R (2.6 ± 1.6 min) was significantly shorter than that for Group P (10.8 ± 3.0 min), with a 95% confidence interval (CI): 6.949-9.431 min, *p* <0.001. Similarly, the time to attain an OAA/S score of 3 was significantly less in Group R (1.6 ± 0.9 min) compared to Group P (9.6 ± 2.6 min), with a 95% CI: 6.930-8.957 min, *p* <0.001.

**Conclusion:** Our study demonstrated that remimazolam anesthesia combined with flumazenil antagonism causes a shorter recovery time for elderly patients undergoing gastrointestinal endoscopy compared to propofol. Remimazolam followed by flumazenil antagonism provides a promising alternative to propofol for geriatric patients, particularly during gastrointestinal endoscopy.

## Introduction

Recovery time is a critical concern for endoscopists and anesthesiologists, as it is imperative to facilitate a prompt and secure recuperation for patients and to maintain the efficiency of endoscopy centers. Recent advancements in medical protocols have positioned propofol as the sedative of choice for endoscopic procedures. This preference is attributed to propofol's advantageous pharmacological profile, which includes a quick onset of action, a short duration of effect and minimal side effects.

Remimazolam is a novel intravenous sedative agent with a highly selective affinity ligand for the benzodiazepine site on gamma-aminobutyric acid (GABA) receptors. Its rapid metabolism into inactive metabolites by tissue esterases ensures an organ-independent metabolic process, making it unaffected by any level of renal impairment and by mild to moderate hepatic impairment 1. Pharmacokinetic and pharmacodynamic models demonstrate that remimazolam exhibits high clearance rates, which prevents accumulation even with continuous infusion. Additionally, its effects can be specifically reversed by flumazenil, further underscoring its safety profile 2.

Due to the use of flumazenil antagonism during gastrointestinal endoscopy in elderly patients in this study, we hypothesized that anesthesia with remimazolam would result in shorter recovery times compared to propofol. The primary goal of this study was to evaluate and compare the recovery time of remimazolam besylate and propofol anesthesia during endoscopic procedures in elderly patients.

## Methods

### Participants

This study received approval from the Clinical Research Ethics Committee of Shidong Hospital in Yangpu District, Shanghai (YPSDKY2022-01-007), and registered by Jin-chao Song on the Chinese Clinical Trial Registry (http://www.chictr.org/cn/) with the registration number ChiCTR2200060474. Following the acquisition of written informed consent, the study encompassed a cohort of 60 elderly patients, aged between 65 and 95 years, with body mass index 19-27 kg/m^2^, and classified under American Society of Anesthesiologists (ASA) physical status I to III. These participants were scheduled for either diagnostic or therapeutic gastrointestinal endoscopy procedures.

Patients presenting known allergy to the drugs under study, a history of chronic usage of sedative or opioid analgesic medications, severe respiratory disease (evidenced by a breath-hold test result of less than 20 seconds) or heart failure (characterized by an ejection fraction of less than 40%) were precluded from participation in this study. Subjects were randomized and allocated equally to either the propofol or the remimazolam group by a computer-generated algorithm. The blinding protocol extended to patients, anaesthetist assistants and gastroenterologists, who were unaware of the group assignments and the trial's objectives. Due to the distinct physical appearances of the study drugs, anesthetist assistants observed and recorded vital signs of patients undergoing gastroenteroscopy from an adjacent room, utilizing a local area network to ensure the integrity of the blinding process.

### Study design and anesthesia protocol

None of the patients received premedication. Upon entering the endoscopy room, a 20-gauge intravenous cannula was placed into a peripheral vein for drug administration and infusion of Ringer Lactate solution. The Philips HP Viridia 24/26 M1205A and the Bene View N15 monitors were employed to continuously monitor the patient's electrocardiogram (ECG), heart rate (HR), non-invasive blood pressure, and peripheral oxygen saturation (SpO_2_) throughout the endoscopic examination. Oxygen was supplied through a nasal catheter at a flow rate of 3-5 liters per minute (L/min) during the procedure. Prior to commencing the procedure, patients were given a 5-minute period to rest. Following this, baseline hemodynamic parameters, including mean arterial blood pressure (MBP) and HR, were measured and recorded. The induction time was defined as the time from the administration of either remimazolam or propofol until an OAA/S score of 1 was achieved, which was also recorded. Additionally, the duration of the endoscopic procedure and any perioperative adverse events associated with anesthesia were meticulously recorded., These events included hypotension, bradycardia, respiratory depression, body movement, postoperative nausea and vomiting (PONV), dizziness, and injection pain.

Following preoxygenation, the participants in the Group R received an infusion of remimazolam (Yichang Humanwell Pharmaceutical Co., Ltd., China) at a rate of 6 mg•kg^-1^•h^-1^ using a Graseby 3500 syringe pump until an OAA/S score of 1was achieved (patients does not respond to mild prodding or shaking). Then, a bolus dose of 5 mg remimazolam was administered to deepen the anesthesia. After allowing one minute for the bolus to exert its effect, the endoscopic procedure commenced. Anesthesia was maintained during the procedure with a continuous infusion of remimazolam at a rate of 1 mg•kg^-1^•h^-1^. In instances of coughing, retching, or purposeful movement of the head or limbs during the endoscopy, a rescue medication of 5 mg remimazolam was given immediately. In our endoscopy centre, patients underwent gastroscopy followed by colonoscopy in one anaesthetic treatment. Upon completion of the endoscopy, 0.2 mg flumazenil was intravenously injected within 15 seconds to reverse the sedative effects of remimazolam as a routine.

In the Group P, propofol was administered at a rate of 18 mg•kg^-1^•h^-1^ until an OAA/S score of 1 was achieved. Then, the endoscopic procedure began, with anesthesia being maintained using propofol at a lower rate (6-9 mg•kg^-1^•h^-1^). Throughout the procedure, anesthesiologists meticulously adjusted the rate of propofol administration to ensure an optimal depth of anesthesia. In the event of coughing, retching, or purposeful movement of the head or limbs, a rescue medication of 20-30 mg propofol was promptly administered.

Emergency equipment and medication were available throughout the gastroenteroscopy procedure. In instances where the MBP decreased to below 60 mmHg, or when the systolic blood pressure (SBP) fell below 90 mmHg, patients were given 5-15 mg of ephedrine or an equivalent dose of metaraminol as warranted. Additionally, a decrease in heart rate to below 50 beats per minute, prompted the administration of 0.25mg atropine. In cases where a patient's SpO_2_ level was below 90%, measures such as assisted mask ventilation were provided as necessary.

Throughout the endoscopic procedure, trained anaesthetist assistants measured and recorded MBP, HR, and SpO_2_ levels of patients at designated time points. The designated time points were defined as: T_0_ = baseline values; T_1_ = when OAA/S score reached 0; T_2_ = at scope intubation and T_3_ -T_10_= by 3-min intervals during the endoscopic procedure.

The primary endpoint was recovery time, which was defined as the time from discontinuing remimazolam or propofol to OAA/S = 5. The duration between discontinuing either remimazolam or propofol and reaching an OAA/S score of 3 was also recorded. The secondary endpoint was the percent change to baseline in MBP and HR. Percent change in MBP = [(MBP_Tx_ - MBP_T0_)/ MBP_T0_] * 100. Percent change in HR = [(HR_Tx_ -HR_T0_)/HR_T0_] * 100. Tx represents the designated time points.

### Statistical analysis

The sample size for this study was established based on the outcomes of a preliminary investigation, which indicated a mean recovery time of 9.0 ± 4.5 (n = 6) for Group P and 4.0 ± 3.0 (n = 6) for Group R. To calculate the required sample size, the following formula was utilized: n = 15.7/ [(difference between groups) / (mean of the SD between groups)]^2^ + 1. This calculation determined that 10 subjects in each group per group would achieve a statistical power of 0.80 and type I error rate of 0.05. Considering the secondary endpoints and referencing previous research of my own 3, 4, the sample size was adjusted to 30 patients per group. Thus, a total of 60 patients will be allocated to the respective groups via a computerized process.

All data in the text and tables were expressed as the mean (standard deviation, SD), median [interquartile range (IQR)], or number of patients (%). Continuous variables with normal distribution were analyzed with independent 2-sample t-test, while skewed distributed continuous variables were analyzed using Mann-Whitney U test. Categorical variables were analyzed using the Pearson chi-square test. *p* values were two-sided, with *p* < 0.05 considered statistically significant. All analyses were performed using SPSS 17.0 (SPSS Inc., Chicago, IL). Figures were made with GraphPad Prism 5.

## Results

**Figure 1:**
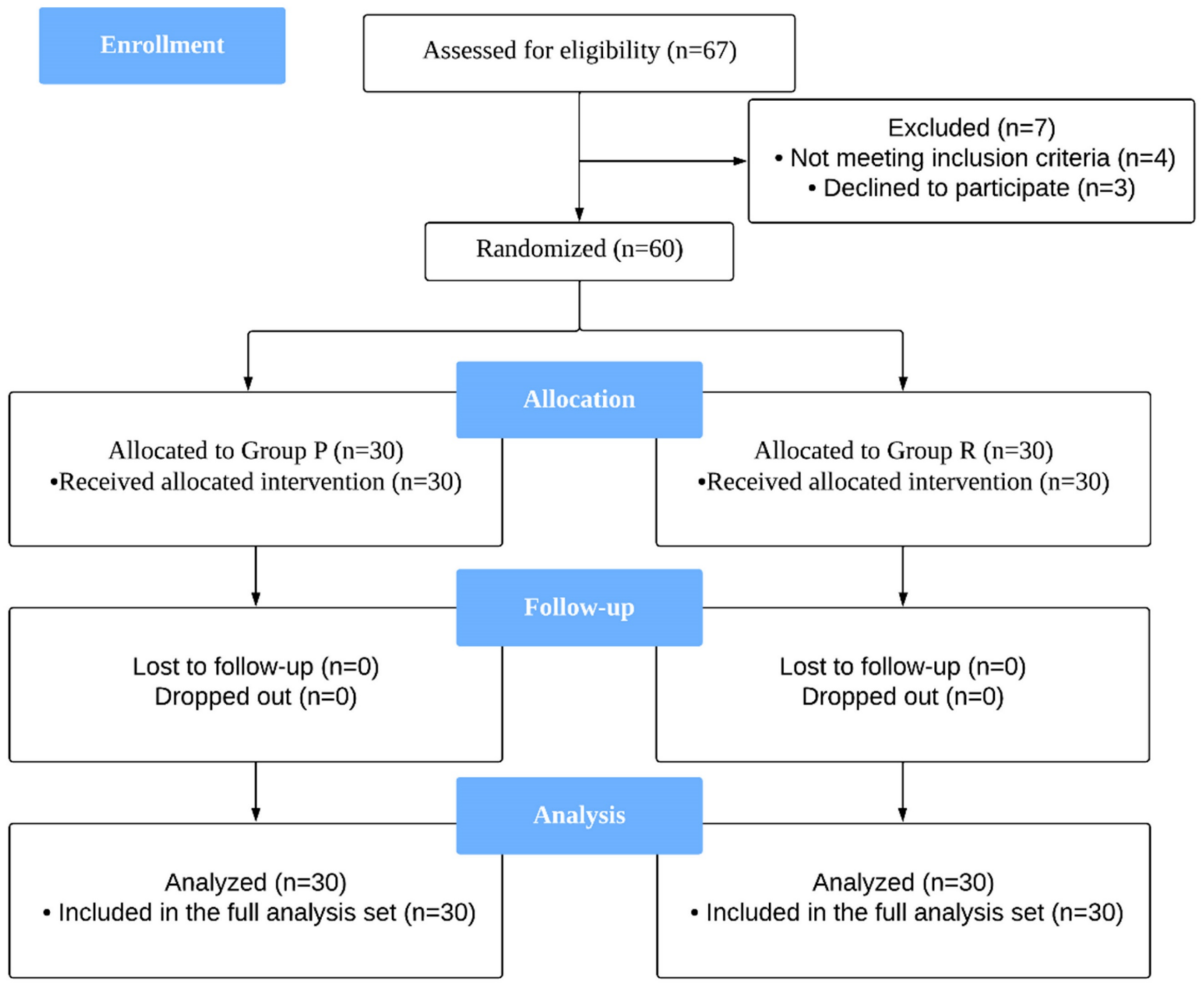
CONSORT diagram describing patient progress through each stage of the randomized trial.

As shown in** Figure 1**, a total of 67 patients were assessed for eligibility; among them, 60 patients who fulfilled the inclusion criteria and provided informed consent were enrolled in the study. These patients were randomly and equally assigned to either Group R or Group P.

The demographic characteristics and perioperative data of the 60 participants in the two groups are presented in **Table 1**. Patients in Group R experienced significantly shorter operation times compared to their counterparts in Group P (*p*<0.001). Furthermore, it was noted that injection pain occurred more frequently in Group P than in Group R (*p*=0.038). Nevertheless, no significant disparities were observed regarding other intraoperative adverse events, including body movements, hypotension and bradycardia. Moreover, our analysis revealed no significant differences between the two groups in terms of age, gender, body mass index (BMI), international normalized ratio (INR), duration of the gastrointestinal endoscopy, postoperative outcomes, and liver and kidney function parameters.

**Table 2** presents the comparison of recovery times, defined as the time from discontinuing remimazolam or propofol to reaching an OAA/S score of 5, between Group P (10.8 ± 3.0 min) and Group R (2.6 ± 1.6 min). The findings revealed that the recovery time for Group R was significantly shorter than that for Group P (95% CI: 6.949-9.431 min, *p* <0.001). Similarly, the time recorded from discontinuing remimazolam or propofol to reaching an OAA/S score of 3 was shorter in Group R (1.6± 0.9 min) compared to Group P (9.6 ± 2.6 min), with statistical significance (95% CI: 6.930-8.957 min, *p* <0.001).

**Figure 2:**
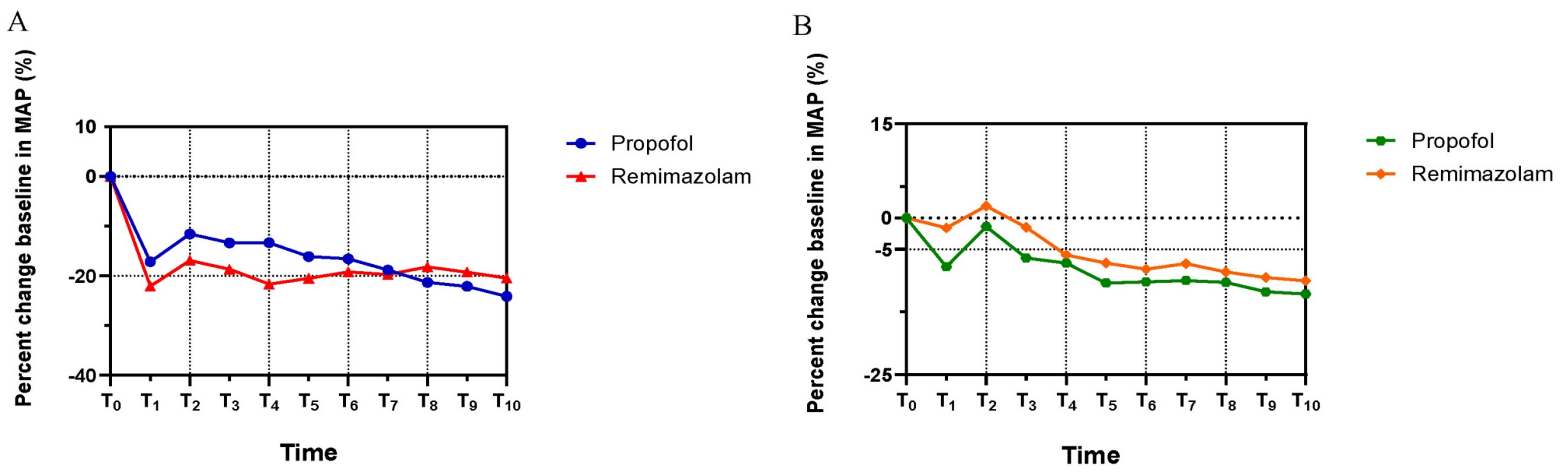
** The trends of patients' hemodynamic profiles relative to baseline during endoscopy.** (A) Mean arterial pressure (MAP), (B) Heart rate (HR). The percent changes of MAP and HR at eleven different time points in each group were compared in Student's t test. All the values are presented as mean. T_0_: Basic hemodynamic parameters of patients; other data were recorded every 3 minutes till the end of endoscopy at T_1_-T_10_.

**Figure 2** shows the MAP trends for both groups, which decreased rapidly until T_1_ (3 min post-anesthetic administration), then increased at T_2_ due to the stimulatory effects of endoscopic intubation, and remained stable until the end of endoscopy. There was no statistically significant difference in MAP between the groups throughout the procedure. (MAP: T_1_, *p*=0.194; T_2_, *p*=0.181; T_3_, *p*=0.167; T_4_, *p*=0.064; T_5_, *p*=0.381, T_6_, *p*=0.608; T_7_, *p*=0.854; T_8_, *p*=0.531; T_9_, *p*=0.575; T_10_, *p*=0.441). Notably, Group R exhibited a marginally higher MAP compared to Group P after T_7_. In addition, the HR in Group R was relatively higher than in Group P from T_1_ to T_10_, decreasing after induction until T_1_ and increasing following the endoscopic intubation stimulus at T_2_ in both groups. No statistically significant difference in HR was observed between the groupsas well (HR: T_1_, *p*=0.075; T_2_, *p*=0.381; T_3_, *p*=0.152; T_4_, *p*=0.710; T_5_, *p*=0.337, T_6_, *p*=0.582; T_7_, *p*=0.447; T_8_, *p*=613; T_9_, *p*=0.502; T_10_, *p*=0.538).

## Discussion

In this study, we investigated the recovery times of elderly patients undergoing gastrointestinal endoscopy with either remimazolam or propofol anesthesia. The findings demonstrated that remimazolam anesthesia caused a significantly shorter recovery time compared to propofol anesthesia due to the use of flumazenil antagonism. No serious complications were observed in either group during the study. Endoscopists and anesthesiologists have commonly expressed concern regarding recovery time, as rapid recovery is of great importance for both patients and endoscopy centers 5, 6. The recovery time is influenced by the pharmacokinetics of the anesthetic used as well as the physiological condition of the patient.

In our pilot study, we observed a certain proportion of body movements in patients who received remimazolam as the single anesthetic drug for sedation during the gastrointestinal endoscopy. Clinical experience suggested that administering a 5 mg bolus of remimazolam after achieving an OAA/S score of 1 could deepen anesthesia and lower the incidence of body movements. Consequently, a 5 mg bolus of remimazolam was administered before commencing the endoscopic procedures.

Propofol is predominantly metabolized through biotransformation into glucuronide conjugates, with uridine diphosphate-glucuronosyltransferases playing a crucial role in its metabolism 7. These enzymes are expressed in both hepatic and extrahepatic tissues 8. Shafer *et al.* reported that the pharmacokinetics of propofol included a total body clearance rate of 2.09 ± 0.65 L/min and an elimination half-life of 116 ± 34 min 9. Studies have consistently shown that clearance rates of propofol in elderly patients are significantly lower than in younger patients, indicating that age is a significant factor affecting propofol metabolism 10, 11.

Remimazolam, acting on GABA receptors, characterized by its fast onset, quick recovery and organ-independent metabolism. Additionally, its sedative and hypnotic effects can be reversed by flumazenil, similar to other benzodiazepines 12. Remimazolam exhibits a limited volume of distribution and maintains a consistent half-life of 6-7 minutes, irrespective of administration duration, body weight, gender, or ethnic-related pharmacokinetic variations. This has been substantiated through both non-compartmental and compartmental modeling techniques, as well as a recirculatory model. Schüttler *et al.* reported that remimazolam has a high elimination clearance (1.15 ± 0.12 L/min) with small interindividual variability, a short terminal half-life (70 ± 10 min) and its context-sensitive half time (CSHT) after a 4-h continuous infusion was predicted to be 6.8 ± 2.4 min 13. Similar results were confirmed by Wiltshire *et al.*, suggesting that remimazolam does not accumulate after constant infusion and can be used for the maintenance of general anesthesia in order to obtain a quick full-alert time 14. Furthermore, renal and hepatic dysfunction do not adversely affect the clearance of remimazolam, owing to its organ-independent metabolic profile. Studies have shown that severe hepatic impairment results in only a minor delay in recovery from remimazolam anesthesia when compared to the propofol anesthesia group, with no significant difference observed between the two groups in the presence of renal impairment 1.

As discussed, both remimazolam and propofol are characterized by short elimination half-life and rapid induction and emergence from anesthesia, reflecting their pharmacokinetic properties and clinical utility. However, whether remimazolam facilitates faster recovery times and superior recovery quality compared to propofol remains a subject of debate 15. In our study, the recovery time of remimazolam anesthesia (2.6 ± 1.6 min) was significantly shorter than that of propofol anesthesia (10.8 ± 3.0 min) through the use of flumazenil antagonism during gastrointestinal endoscopy in elderly patients. These results were consistent with a study by Chen *et al.*, which showed a 13.5% reduction in the recovery time of remimazolam anesthesia compared to propofol anesthesia 16. A recent study demonstrated that incorporating flumazenil into a remimazolam-based total intravenous anesthesia regimen significantly expedited recovery of consciousness with reduced variability compared to propofol anesthesia in patients undergoing open thyroidectomy. In this research, patients receiving remimazolam combined with flumazenil for their anesthesia regimen experienced a faster and more consistent recovery of consciousness than those administered propofol 17. However, a previous study showed no significant difference in recovery time between remimazolam or propofol for general anesthesia, with both having mean recovery times of 7.1 min and 8.1 min, respectively 18. This suggests that the use of flumazenil antagonism as a routine upon cessation of the procedure by the gastrointestinal endoscopist may significantly contribute to the accelerated recovery observed in our study. Moreover, we recorded the time from discontinuing remimazolam or propofol to reaching an OAA/S score of 3. It was found that patients received remimazolam anesthesia (1.6 ± 0.9 min) reached this level of alertness significantly faster compared to those who received propofol anesthesia (9.6 ± 2.6 min). This finding indicates that an anesthesia regimen combining remimazolam with flumazenil facilitates a comparatively faster "awakening" process than propofol anesthesia. It suggests that patients receiving the remimazolam-flumazenil combination tend to respond to loud or repeated calls and open their eyes sooner than those administered propofol, until they achieve full consciousness.

The hemodynamic profile of remimazolam has been shown to be more stable compared to propofol. Unlike propofol, remimazolam does not exhibit a dose-dependent reduction in systemic vascular resistance or a dose-dependent decrease in cardiac contractility. Urabe *et al.* identified a potential mechanism where remimazolam may reverse the increase in calcium concentration in endothelial and neuronal cells through the G-protein coupled receptors (GPCRs)-inositol 1,4,5-trisphosphate (IP3) pathway, a phenomenon not observed with propofol hemodynamics 19. In our study, both groups exhibited a decline in MAP and HR following continuous infusion of remimazolam or propofol until T_1_. Subsequent to endoscopic intubation at T_2_, both MAP and HR increased and then stabilized. Notably, patients in Group R experienced a gradual increase in MAP over time that surpassed that of the Group P. In addition, the HR of patients in Group R was slightly higher than that of patients in Group P after induction. These findings suggest that remimazolam may have a less significant impact on hemodynamics compared to propofol, corroborating previous research that posits remimazolam as a safer alternative for inducing anesthesia in high-risk patients 13, 20. Furthermore, studies have indicated that remimazolam causes fewer intraoperative adverse events compare to propofol, including hypotension and injection pain 21-23. Our study found that the injection pain reported by participants in Group R was significantly lower than that reported by those in Group P.

There are several limitations in this study that warrant mention. First, it is crucial to include a larger cohort to definitively establish whether there is a significantly higher incidence of perioperative adverse events in the Group P compared to the Group R, and to ascertain the minimal effective doses of remimazolam for elderly patients. Second, our study primarily focused on recovery time; future research should examine indices of recovery quality. Such investigations could provide critical insights into postoperative cognitive dysfunction among elderly patients. Lastly, the inclusion of a broader array of clinical procedures is essential, as the recovery times associated with remimazolam anesthesia require further validation.

**In conclusion**, our study demonstrated that remimazolam anesthesia combined with flumazenil antagonism causes a shorter recovery time for elderly patients undergoing gastrointestinal endoscopy compared to propofol. It is recommended that the intravenous administration of 0.2 mg of flumazenil within a 15-second window as a routine immediately upon cessation of the procedure by the gastrointestinal endoscopist can effectively reverse the sedative effects of remimazolam. This approach offers a promising alternative to propofol for geriatric patients, particularly during gastrointestinal endoscopy.

## Figures and Tables

**Table 1:** Demographic characteristics and Perioperative data.

	Group P (n=30)	Group R (n=30)	*P* value
Age, y	69.5 [66.0-72.0]	68.0 [67.0-72.3]	0.789
Male, n (%)	9 (30)	11 (36.7)	0.584
BMI, kg/m^2^	23.3 (3.3)	23.0 (2.0)	0.652
INR	0.92 (0.04)	0.90 (0.05)	0.262
Liver function			
TBil, μmol/L	15.0 (3.8)	14.5 (3.6)	0.719
DBil, μmol/L	2.4 (0.7)	2.8 (0.6)	0.139
ALb, g/L	41.0 (3.3)	41.5 (4.0)	0.698
ALT, U/L	20.1 (7.7)	21.5 (11.2)	0.685
AST, U/L	22.6 (7.3)	22.8 (8.5)	0.949
Kidney function			
Scr, μmol/L	65.3 (13.5)	65.0 (17.4)	0.947
BUN, mmol/L	5.5 (0.9)	5.3 (2.0)	0.773
Dose of anesthetic drug for induction, mg			-
Propofol	115.9 (24.1)	-	
Remimazolam	-	28 (4.6)	
Total dose of anesthetic drug, mg			-
Propofol	409.1 (161.4)	-	
Remimazolam	-	61.4 (17.9)	
Duration of anesthetic induction, min	6.2 (1.3)	3.9 (0.8)	<0.001^***^
Duration of gastrointestinal endoscopy, min	31.0 (16.3)	28.2 (14.4)	0.489
Intraoperative adverse events, n (%)			
Injection pain,	4 (13.3)	0 (0)	0.038^*^
Body movement	5 (16.7)	9 (30)	0.222
Hypotension	3 (10)	2 (6.7)	0.640
Bradycardia	1 (3.3)	1(3.3)	1.000
Postoperative outcomes, n (%)			
PONV	0 (0)	0 (0)	-
Dizziness	1 (3.3)	0 (0)	0.313
Endoscopic therapeutic procedures, n			
No treatment	7 (23.3)	5 (16.7)	
APC	4 (13.3)	5 (16.7)	
APC+EMR	19 (63.3)	20 (66.7)	

BMI, body mass index; INR international normalized ratio; TBil, total bilirubin; DBil, direct bilirubin; ALb, albumin; ALT, alanine aminotransferase; AST, aspartate aminotransferase; Scr, serum creatinine, BUN, blood urea nitrogen. PONV, Postoperative nausea and vomiting. APC, Argon Plasma Coagulation.; EMR, Endoscopic Mucosal Resection. Data are presented as the mean (SD), median [interquartile range], or number of patients (%). *p* value in Student's t test, Mann Whitney U test or Pearson chi-square test. *p* <0.05, *; *p* <0.01, **; *p* <0.001, ***

**Table 2:** Recovery time of different anesthetic drugs.

Time recorded from discontinuing remimazolam or propofol	Propofol	Remimazolam	*P* value	Mean differences (95% CI)
Time to reach OAA/S score = 3, min	9.6 (2.6)	1.6 (0.9)	<0.001^***^	6.930-8.957
Time to reach OAA/S score = 5, min	10.8 (3.0)	2.6 (1.6)	<0.001^***^	6.949-9.431

OAA/S, Observer's Assessment of Alertness and Sedation scale. Patients can respond after name spoken loudly or repeatedly along with glazed marked ptosis when OAA/S = 3; the recovery time was defined as the time from discontinuing remimazolam or propofol to reaching OAA/S = 5, where patients can respond readily to name spoken in normal tone. *p* value in Student's t test, *p* <0.05, *; *p* <0.01, **; *p* <0.001, ***
